# Arthroscopic Osteochondral Autograft Transplantation (OAT) in Patients with Focal Osteochondral/Chondral Lesions of the Knee Mid-Term Clinical Outcome

**DOI:** 10.5704/MOJ.2507.004

**Published:** 2025-07

**Authors:** N Mehta, J Kumar, A Chopra, A Goyal, D Joshi

**Affiliations:** Department of Sport Injury Centre, Vardhman Mahavir Medical College and Safdarjung Hospital, New Delhi, India

**Keywords:** focal osteochondral lesion, osteochondral autograft transplantation (OAT), knee chondral lesion

## Abstract

**Introduction:**

Articular cartilage has limited healing potential as it is a hypocellular and avascular structure, hence it is to manage articular cartilage defects. The arthroscopic osteochondral autograft transplantation procedure is minimally invasive and cosmetically acceptable procedure to manage cartilage defects.

**Materials and Methods:**

This is a prospective study extending from 2018 to 2023 done at Sports Injury Center, New Delhi involving 36 patients with focal full-thickness chondral/osteochondral defect, age <45 were included. Global Chondral change, Multi ligamentous injuries, varus or valgus malalignment, kissing lesion and defect > 20mm were excluded. The osteochondral lesion was debrided down till healthy cartilage margins, donor graft was harvested from the non-weight-bearing area of the MFC. Lysholm score was used to assess functional outcome.

**Results:**

Median age 35 years (range 20 – 44 years). A total of 51% of the patients were aged between 31 and 40 years (n=18). Male to female ratio was 3.37:1. The median defect size was 8mm (range 7-10mm). There was a gradual improvement in knee-specific symptoms with time. There was a significant increase in Lysholm score with time (p<0.0001).

**Conclusion:**

The OATS procedure is a reliable, reproducible method and its results are encouraging with early mid-term follow-up; however, a long-term follow-up study is required to ascertain the validation of OATS procedure for preventing degenerative arthritis in patients with osteochondral injuries of the knee.

## Introduction

It is a therapeutic challenge to manage articular cartilage defects. Articular cartilage has limited healing potential as it is a hypocellular and avascular structure^1^. Patients with focal chondral lesions of the knee have quality of life like a patient with high-grade osteoarthritis or torn anterior cruciate ligament^1^.

For articular cartilage defects, treatments such as Microfracture, subchondral drilling, abrasion chondroplasty, osteochondral autograft transfer system (OATS), Osteochondral Allograft Transplantation, and autologous chondrocyte implantation (ACI) have evolved in the last two decades^2,3^. OATS is the replacement of cartilage from non-weight-bearing areas of the knee joint to the focal defect in the weight-bearing area^3,4^. OATS technique was popularised by Hangody *et al*^5^.

OATS can be done as an arthroscopic or open procedure. The first arthroscopic-assisted OATS procedure was reported by Matsusue *et al*^6^. The arthroscopic OATS procedure is minimally invasive and cosmetically acceptable but is technically demanding and has a learning curve.

The efficacy of OATS, both open or arthroscopic procedures for treating focal cartilage defects has been documented in the literature but none of them evaluated mid-term outcomes >5years in Asian population^7-12^. This study prospectively evaluates midterm (till 5 years) results of arthroscopic knee OATS procedure.

## Materials and Methods

This is a prospective study extending conducted from 2018 to 2023 at Sports Injury Center, New Delhi with the following inclusion criteria; (1) Age less than 45 years having an active lifestyle with involvement in recreational sports, (2) Focal full-thickness chondral/osteochondral defect diagnosed on MRI, (3) full- thickness chondral/osteochondral defect confirmed by arthroscopy for OATS procedure, (4) symptomatic for more than six weeks which may or may not be associated with trauma. Informed consent was taken from every patient.

Patients with (1) generalised chondral change with space narrowing (>4mm) on weight-bearing anteroposterior radiograph films, (2) multi ligamentous injuries, (3) varus or valgus malalignment (more than 5°), (4) more than one lesion or kissing lesion, (5) defect more than 20mm, and (6) non-compliant to rehabilitation protocol were excluded.

Clinical outcome was evaluated by Lysholm score (which includes limping, mechanical locking, instability, pain, swelling, stair climbing, and squatting) and return to pre-injury activity level. Pre-operative and post-operative data was collected at 3, 6, 12 months and then every year.

For surgical techniques, all patients were operated under spinal anaesthesia and a tourniquet. The standard arthroscopic evaluation was done to assess the concomitant ligaments and menisci injuries. If any ligament or meniscus injury was diagnosed, it was managed first and then the OATS procedure was performed. The osteochondral lesion was debrided down till healthy stable cartilage margins. The area of the lesion was calculated in mm^2^ and then the appropriate OATS set was used.

**Fig. 1:**
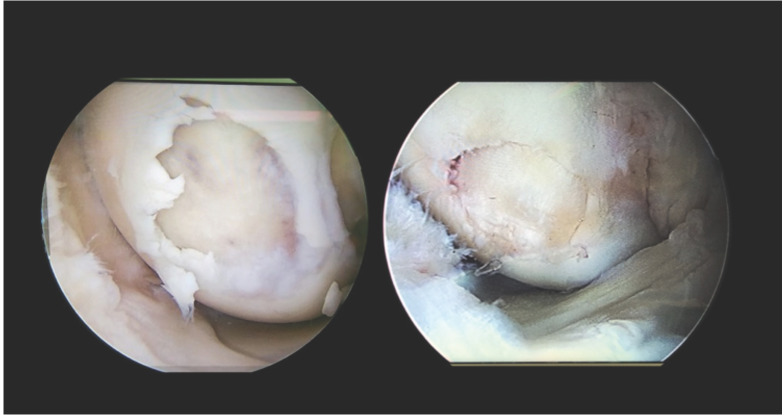
Arthroscopic image showing osteochondral defect filled using OAT Plug.

The donor graft was harvested from the non-weight-bearing area of the medial femoral condyle (MFC). Preparation of the recipient site is done by harvesting the plug which is 5mm smaller than the donor plug. Donor plug placement was done perpendicular to the osteochondral defect of articular surface and plug should not protrude >1mm to ensure a regular smooth surface. The bony plug harvested from the recipient area was tapped back into the donor area (Fig. 1).

Post-surgery no substance such as hyaluronic acid (HA) or Platelet rich plasma (PRP) injection are used in current study and no specific analgesia were given to patients.

For rehabilitation, all the patients underwent similar rehabilitation protocols. The patients were mobilised with crutches and kept non-weight-bearing with locked braces and aimed to achieve quadriceps control, passive full knee extension, and gradual knee flexion in 0-6 weeks.

In 6-12 weeks, patients were allowed to bear weight up to 50% with crutch. After 10-12 weeks full weight bearing was allowed as the patient tolerated. Muscle strengthening and endurance exercises (squats, lunges, cycling) were gradually started after 12 weeks^13^.

After 26-52 weeks all patients (20 – 44 years) returned to recreational sport activities or full functional activities were started and assessed on strength (85% of Isokinetic strength must be achieved compared to normal side). Agility test (T-test must be completed in <11s), and balance (Hop test: limb symmetry index LSI = Distance covered by affected limb / Distance covered by normal limb LSI >0.85).

## Results

The study included 36 patients with median age 35 years and range between 20-44 years. Approximately 51% of the patients were aged between 31 and 40 years (n=18) while 14.3% were above 40 years. Male to female ratio was 3.37:1. Ten patients had ACL tears while five patients had medial meniscus tears. The median defect size was 8mm (range 7-10mm). Two patients were lost to follow-up. The demographic description is given in Table I.

**Table I:** Demographic characteristics.

	n (%)
Age (years)	
11-20	6 (16.7%)
21-30	6 (16.7%)
31-40	18 (51.4%)
>40	5 (14.3%)
Sex	
Male	27 (77.1%)
Female	8 (22.9%)
Concomitant injury	23 (63.9%)
ACL tear	10
Lateral meniscus tear	2
Medial meniscus tear	5

For outcome assessment, the functional outcomes were assessed by Lysholm score. Lysholm score includes the patient's knee-specific symptoms such as pain, swelling, mechanical locking, instability, climbing stairs and squatting. There was a gradual improvement in knee-specific symptoms with time (Table II). There was a significant increase in Lysholm score with time (P<0.0001) (Table III).

**Table II:** Lysholm score.

	Excellent	Good	Fair	Poor
Pre-operative	0	0	0	36
3-months	0	1	22	13
6-months	2	15	19	0
1-year	20	14	2	0
2-year	28	6	0	0
3-year	28	6	0	0
4-year	28	6	0	0
5-year	28	6	0	0

**Table III:** Comparison of Lysholm score with time.

	Lysholm score	P-value
Pre-operative	33.5±9.9	
3-months	66.9±8.6	<0.0001
6-months	78.7±8.4	<0.0001
1-year	85.5±8.6	<0.0001
2-year	88.7±8.8	<0.0001
3-year	89.2±8.1	<0.0001
4-year	94.2±2.9	<0.0001
5-year	93.0±4.2	<0.0001

## Discussion

Articular cartilage defects may spontaneously heal with fibrocartilage. Procedures such as debridement^14^, abrasion arthroplasty, subchondral drilling^15,16^, and microfracture^17^ also promote the formation of fibrocartilaginous tissue, which is biomechanically and histologically inferior to hyaline cartilage^18^.

Horas *et al*^19^ performed a prospective study in patients (n=40) with femoral condyle articular cartilage lesion and were randomly treated with either OATS or ACI to evaluate the 2-year outcomes. Recovery after osteochondral transplantation was faster as compared to ACI. Histologically ACI-treated defects healed primarily with fibrocartilage, whereas hyaline cartilage with osteochondral autograft transplants (OATS), although a persistent interface was present between the graft and the surrounding original cartilage.

OATS procedure helps to cover the chondral defect with hyaline cartilage with an adequate thickness which closely reproduces the curvature of the anatomical femoral condyle. The press-fit technique increases the stabilisation of the grafts in the recipient area which allows for satisfactory initial graft stability without the need for any type of internal fixation.

Bone healing at the interface between the graft and recipient area ensures a higher rate of successful graft incorporation. Better relief of symptoms improved functional outcome, and satisfactory survival of the transplanted hyaline cartilage have been reported by several authors^20-26^.

Yoshizumi *et al* reported successful union with OATS procedure in three patients with osteochondral defects within six months^27^. Jakob *et al* evaluated knee osteochondral autografts with an average follow-up of 37 months in 52 consecutive patients. At 2 years follow-up 86% of patients had improved function of the knee, while 92% of patients observed good to excellent knee function at final follow-up. For full-thickness osteochondral defects treatment, they concluded that OATS is a valid option^28^. In the present study with a 60-month follow-up, a statistically significant improvement in the functional outcome of the knee was observed.

Hangody *et al*^5^ described the results of autologous osteochondral mosaicplasty in 831 patients after 10 years. The patient population consisted of those who had osteochondral transfer to the femoral condyles, tibial, patella/trochlea, and talus. A total of 92% of patients treated with femoral osteochondral transfer had good to excellent results. According to study osteochondral mosaicplasty is a valuable alternative for small to medium-sized focal chondral defects of the weight-bearing area of the knee.

The present study demonstrates the mid-term efficacy of OATS procedure in focal osteochondral defects of the knee. Our clinical outcome assessment provides an average 60-month follow-up for patients with good results.

As there was no major complication, hence re-look arthroscopy was not performed in patients. The present study observed that neither age nor defect size correlates with poor outcomes or results. Hence, OATS procedure is effective in treating full-thickness chondral defects. The results of the present study are comparable with those reported in the literature regarding pain relief with functional improvement.

## Conclusion

The present study is the clinical outcome of the treatment of focal osteochondral defects of the knee with or without other concomitant injuries. The limitation of the study is a small number of patients (n=36) and no control group for comparison. Although follow-up is longer than some published studies, it is not a long-term evaluation. The OATS procedure is a reliable reproducible method, and its results are encouraging with early mid-term follow-up; however, a long-term follow-up study is required to ascertain the validation of OATS procedure for preventing degenerative arthritis in patients with osteochondral injuries of the knee.
